# RAB5A in triple-negative breast cancer: a critical role in macrophage reshaping in an exosomal miR-21-dependent manner

**DOI:** 10.1530/ERC-23-0257

**Published:** 2024-04-12

**Authors:** Lei Qiao, Chao Dong, Wenlei Jia, Gang Sun

**Affiliations:** 1 Department of Breast and Thyroid Surgery, Xinjiang Medical University Affiliated Tumor Hospital, Urumqi, Xinjiang Uygur Autonomous Region, China

**Keywords:** RAB5A, macrophage polarization, exosome, miR-21, triple-negative breast cancer

## Abstract

Breast cancer is the leading cause of cancer-related deaths in females, and triple-negative breast cancer (TNBC) is characterized as one of the main subtypes of breast cancer, with poor prognosis and limited treatments. Investigating the molecular basis or discovering relevant oncogenes will greatly help in developing effective targeted therapies. In this study, we ascertained that RAB5A depletion in TNBC cells suppresses the secretion of exosomes and blocks the polarization of macrophages toward an M2 phenotype. By scanning miRNAs associated with macrophage polarization, we identified that miR-21 was the pivotal component in tumor cell-derived exosomes and played a key role in RAB5A-mediated macrophage polarization. The enhanced expression of miR-21 in macrophages is able to potentiate the M2 polarization of macrophages in the presence of tumor cells. Pellino-1 (PELI1) was subsequently identified as the target of miR-21, and forced PELI1 expression partially abrogated the M2 polarization of macrophages induced by miR-21 overexpression. Macrophages stimulated with RAB5A-depleted TNBC cells (coculture, conditioned medium or exosomes) impaired their capability to promote the proliferation, migration, and invasion of tumor cells. *In vivo* xenograft experiments further confirmed that RAB5A knockdown TNBC cells exhibited reduced tumor formation and impaired tumor-associated macrophage recruitment. These studies shed light on the potential underlying mechanism of RAB5A-mediated macrophage polarization in an exosomal miR-21-dependent manner and provide an experimental basis for the development of RAB5A- or exosome-based tumor therapeutic strategies.

## Introduction

The global cancer burden released by the International Agency for Research on Cancer (IARC) in 2020 shows that there are 2.26 million new cases of breast cancer worldwide, making it the most commonly diagnosed cancer worldwide. It is estimated that the number will be 2.9 million in 2022 (Siegel *et al.* 2021). Additionally, breast cancer was the leading cause of cancer-related deaths in females worldwide in 2020 (680,000 cases). In China, approximately 420,000 cases were newly diagnosed with breast cancer in 2020 (Wild *et al.* 2020). Triple-negative breast cancer (TNBC) is a common subtype of breast cancer, accounting for 15% of all breast cancers (Lin *et al.* 2012). However, it is recognized as the most malignant and aggressive subtype of breast cancer, clinically defined by negative estrogen receptor, progesterone receptor, and human epidermal growth factor receptor-2 expression (Troester & Swift-Scanlan 2009, Singh *et al.* 2020). Surgery is the preferred strategy for TNBC, while for those with inoperable or metastatic TNBC, few effective targeted therapeutic strategies are available due to the lack of specific drug targets (Engebraaten *et al.* 2013). First-line chemotherapy treatments for TNBC include gemcitabine, nab-paclitaxel, and doxorubicin, but their efficacy is limited (Dietze *et al.* 2015). Besides, TNBC frequently develops chemoresistance. Upon chemotherapeutic exposure, cancer cells are able to maintain their survival through various approaches, including inducing cellular senescence or autophagy, altering drug uptake or efflux, activating cancer stem cells, strengthening DNA damage repair, inactivating apoptosis, etc. (O’Reilly *et al.* 2015, Nies *et al.* 2015, Nath *et al.* 2022).

Exosomes are extracellular vesicles (diameter 30–150 nm) secreted by cells with various biological functions (Mears *et al.* 2004, Sharma *et al.* 2016). Their involvement in intercellular communication has been widely depicted by delivering signaling molecules, including mRNAs, miRNAs, sRNAs, proteins, lipids, and metabolites, to recipient cells, leading to the alteration of the functional properties of the recipient cells (Mittelbrunn & Sánchez-Madrid 2012, Tai *et al.* 2018). Despite the ubiquitous secretion of exosomes by most types of cells, malignant cells usually release more exosomes than their normal counterparts (Tickner *et al.* 2014). It has been well established that exosomes released by cancer cells stimulate growth and migration, modulate immune cell responses, and facilitate cancer progression and metastasis (Iero *et al.* 2008, Zhang & Grizzle 2014). When stimulated by microenvironmental signals, e.g. cytokines or microbial products, macrophages tune their functional properties to play different roles (Mantovani *et al.* 2002). Tumor-associated macrophages, including classically activated macrophages (M1) and selectively activated macrophages (M2), are the most predominant leukocyte population in the microenvironment of primary and metastatic tumors, with anti- or protumoral functions (Hanahan & Weinberg 2011, Quatromoni & Eruslanov 2012). M1 macrophages are capable of producing and secreting IL-1β, IL-6 TNF-α, iNOS, nitric oxide, etc., thus exhibiting a proinflammatory and antitumor phenotype (Atri *et al.* 2018). In contrast, TGF-β, IL-10, and chemokines CCL17 and AMAC-1 produced by M2 macrophages are anti-inflammatory and play a role in tumor progression or metastasis (Mantovani *et al.* 2017). An increase in M2 macrophages has been identified as a hallmark of poor prognosis and metastasis in several cancers (Cao *et al.* 2015, Jackute *et al.* 2018). Tumor-derived exosomes, in principle, are capable of reprogramming the macrophage phenotype to provide a microenvironment that favors tumor growth and metastasis (Su *et al.* 2016, Pritchard *et al.* 2020, Li *et al.* 2022).

microRNAs (miRNAs) are a class of small non-coding single-stranded RNA, usually composed of 19–25 nucleotides. Mature miRNAs suppress the expression of targeted genes by directly binding to the 3′-untranslated region (3′-UTR) of the target mRNA, blocking translation, or causing the degradation of the mRNA (Krol *et al.* 2010). Previous studies have shown that as an important gene regulator, miRNA widely participates in various biological processes, such as cell proliferation, cell cycle regulation, apoptosis, migration, and invasion (Bartel 2004, Ashrafizadeh *et al.* 2020*b*). Subsequent studies have found that miRNA is dysregulated in various cancers and plays roles as either oncogenes or tumor suppressors, which are tightly related to tumor development and occurrence (Calin & Croce 2006), as well as chemoresistance (Ashrafizadeh *et al.* 2020*a*).

Rab5 belongs to the Rab GTPase family, and three isoforms have been identified, RAB5A, RAB5B, and RAB5C (Bucci *et al.* 1995, Pfeffer & Aivazian 2004). The aberrant expression of RAB5A has been widely documented in various human tumors, e.g. breast cancer, lung cancer, and ovarian cancer; furthermore, the expression of RAB5A is tightly associated with tumorigenesis and metastasis (Yu *et al.* 1999, Su *et al.* 2007, Zhao *et al.* 2010, Yang *et al.* 2011). In the current study, we revealed that RAB5A engages in the secretion of exosomes in TNBC cells, and exosomes derived from RAB5A-deficient cells cause the repolarization of tumor-associated macrophages by regulating miR-21. Our results provide new insight into RAB5A-mediated TNBC progression.

## Materials and methods

### Cell lines and culture

MDA-MB-231 cells and THP-1 cells (the human monocytic cell line) were obtained from Procell Life Science & Technology Co., Ltd. (Wuhan, China), and MDA-MB-468 cells from the American Type Culture Collection were grown in Dulbecco’s modified Eagle’s medium (DMEM) supplemented with 10% fetal bovine serum and 1% penicillin‒streptomycin (Invitrogen) at 37°C. Upon reaching 80–90% confluence, cells were passaged at a ratio of 1:5–1:10, and the following assays were conducted with cells in the logarithmic growth phase. The differentiation of THP-1 cells was induced by incubation with 50 ng/mL phorbol 12-myristate 13-acetate (PMA) for 48 h.

### Cell transfection

Small interfering RNAs targeting RAB5A were generated in our previous report (Qiao *et al*. 2023; siNC, 5′-UUCUCCGAACGUGUCACGUTT-3′ and 5′-ACGUGACACGUUCGGAGAATT-3′; siRAB5A#2, 5′-CCAGUUCAAACUAGUACUUTT-3′ and 5′-AAGUACUAGUUUGAACUGGTT-3′). Cells were seeded in a 24-well plate (5 × 10^4^ cells/well), and 50 μL of Block-iT Alexa Fluor Red (Thermo Fisher), lipofectamine RNAiMAX (Thermo Fisher), and siRNA or vectors were added to the plate and incubated at 37°C for 24 or 48 h.

### Conditioned medium preparation and cell treatment

MDA-MB-231 and MDA-MB-468 cells with indicated transfections were plated in DMEM medium (10% fetal bovine serum) and cultured for 48 h. The medium was collected and centrifuged at 4°C, 300 ***g*** for 10 min to remove the cell debris, and the supernatant was collected for further use. The PMA-pretreated THP-1 cells were incubated with an equal volume of conditioned medium derived from TNBC cells. For exosome incubation, the isolated exosomes were first quantified and diluted into the equal concentrations. PMA-pretreated THP-1 cells were plated in DMEM medium, and an equal volume of exosomes was added to the culture.

### RNA extraction and quantitative reverse transcription-PCR (qRT‒PCR)

Cells were homogenized in TRIzol reagent at 4°C for 5 min. Cell homogenates were then transferred to RNase-free tubes, mixed with chloroform (1/5 TRIzol volume), and vibrated for 15 s. After centrifugation at 14,000 ***g*** for 15 min, the upper phase was carefully transferred to a new tube, and an equal volume of isopropanol was added. The mixture was centrifuged, and the supernatant was removed. The precipitate was rinsed with 75% ethanol and redissolved using DEPC water. The isolated RNA was subsequently quantified using a NanoDrop 2000 and stored at −80°C for further use.

A 5× All-In-One RT MasterMix kit (Applied Biological Materials, Canada) was employed for cDNA synthesis. The reverse transcription reaction was conducted at 37°C for 15 min, followed by 60°C for 30 min, and stopped at 85°C for 3 min. The synthesized cDNA was preliminarily quantified with Qubit fluorometer using Qubit™ ssDNA kit (Thermo Fisher). The quantification was performed with purified cDNA using QIAquick columns (Qiagen). One microliter cDNA (~20 ng) was subjected to qRT-PCR analysis with EvaGreen Express 2× qPCR MasterMix-Low Rox (Applied Biological Materials) on an ABI QuantStudio 6 Flex Real-Time PCR System (Applied Biosystems). The primers used in this study were as follows: pri-miR-21 F: 5′-TGTTTTGCCTACCATCGTGA-3′, R: 5′-AAGTGCCACCAGACAGAAGG-3′; premiR-21 F: 5′-GCTTATCAGACTGATGTTGACTG-3′, R: 5′-CAGCCCATCGACTGGTG-3′; GAPDH F: 5′-ACCCAGAAGACTGTGGATGG-3′, R: 5′-CAGTGAGCTTCCCGTTCAG-3′. Other primer sequences used in this study are in Supplementary Table 1 (see section on supplementary materials given at the end of this article). The expression of mature miRNAs was determined using a TaqMan miRNA assay in which RNUB6 was employed as the internal control.

### Western blot

Cells were first rinsed with PBS three times, and 100 μL lysis buffer (RIPA:PMSF = 100:1, v/v) was added to the cells followed by vibration for 5 min. The mixture was kept on ice for 30 min and centrifuged for 20 min at 14,000 ***g*** and 4 °C. The supernatant was collected and quantified using a BCA kit (Beyotime, Shanghai, China) according to the manufacturer’s instructions. The protein was denatured by boiling for 5 min with 5× SDS loading buffer. Approximately 20 μg protein were separated with SDS-PAGE (120 V, ~60 min) and transferred to PVDF membranes (Millipore) at 4°C for 1.5 h. After blocking with 5% skimmed milk at room temperature for 2 h, the membranes were incubated with primary antibodies at 4°C overnight and subsequently incubated with secondary antibody at 37°C for another hour. After washing with TBST three times, the blots were visualized with SuperSignal West Pico PLUS Chemiluminescent Substrate (Thermo Fisher).

### Exosome isolation

Cell culture medium (30 mL) was collected and subsequently centrifuged at 10,000 ***g*** for 30 min and filtered through a 0.22 µm filter to remove cellular fragments and oversized extracellular vesicles. Samples mixed with an equal volume of XBP buffer were transferred to an exoEasy spin column and centrifuged at 5000 ***g*** for 1 min. After discarding the eluate, the spin column was washed using 10 mL XWP buffer. The exosomes remaining on the spin column were subsequently eluted using 400 μL XE buffer.

### Transmission electron microscopy

Extracted exosomes were fixed with 2.5% glutaraldehyde, and one drop of exosomes was placed on a copper grid and negatively stained using 2% phosphotungstic acid for 2 min. The excess staining solution was carefully removed, and the copper grid was dried at room temperature. The exosomes were observed under a Tecnai G2 F30 at 100 kV.

### Nanoparticle tracking analysis

The isolated exosomes were resuspended in PBS and homogenized, followed by filtration through a 0.22 μm filter. The exosomes were tested using NanoSight NS300 (Malvern Instruments, Malvern, UK), and the results were analyzed using NTA 2.3 Analytical Software (Malvern Instruments). The tests were repeated three times for each sample.

### Internalization of PKH26-labeled exosomes into THP-1 cells

The isolated exosomes were mixed with PKH26 fluorescent dye (#UR52302, Umibio, Shanghai, China) and incubated at 37°C for 10 min in the dark. PBS mixed with fluorescent dye was served as the negative control. An equal volume of 1% BSA was added to neutralize the excessive dye. The mixture was centrifuged at 100,000 ***g*** for 60 min and resuspended with PBS. THP-1 cells (1 × 10^4^) were incubated with labeled exosomes for 6 and 12 h, subsequently fixed with 4% paraformaldehyde for 30 min at room temperature. The cell nuclei were counterstained with DAPI for 10 min. The fluorescent signals were visualized under a Leica SP8 confocal microscope (Leica).

### Luciferase reporter assay

A fragment of the 3′-UTR of Pellino-1 (PELI1) harboring the binding site of miR-21 was inserted into a pMIR-REPORT luciferase vector (WT luciferase vector), while the MUTANT luciferase vector was generated by inserting a mutated binding site instead. The luciferase reporter assay was performed using a dual luciferase kit (Promega). The β-gal reporter vector (β-gal) was used as the internal reference.

Cells at 70–80% confluence were transfected with miR-21 mimics, along with luciferase and β-gal vectors. After incubation for 48 h, the cells were lysed, and the luciferase activity and β-gal activity were immediately detected according to the instructions of the manufacturer.

### CCK-8 assay

Cells with the indicated treatments were added to 10 µL CCK-8 reagent (Dojindo, Japan) and incubated at 37°C for 2 h. The absorbance was then detected at 450 nm.

### Wound healing assay

Trypsinized cells (5 × 10^5^) were seeded on six-well plates (supplemented with 10% FBS) and incubated at 37°C for 24 h. A wound scratch was created using a sterilized pipette tip, and DMEM without FBS was added. The cells were incubated at 37°C for 24 h. The scratch was photographed at 0 h and 24 h.

### Transwell invasion assay

The Transwell insert was first coated with Matrigel and seeded with 1 × 10^5^ cells in the upper chamber, and the lower chamber was supplemented with DMEM (10% FBS). After incubation at 37°C for 24 h, the insert was fixed with methanol for 30 min and stained with crystal violet for another 15 min. The cells remaining on the upper surface were carefully removed using a cotton swab. The invaded cells on the lower surface were counted under a light microscope.

### Apoptosis assay

The apoptosis of cells was performed using the Annexin V FITC Apoptosis Detection Kit (BD Bioscience) according to the instructions of manufacturer. Briefly, cells were trypsinized for 2–3 min, washed with PBS, and resuspended in 1× binding buffer. Subsequently, cells were mixed with 5 μL annexin V-FITC and 5 μL PI-PE and incubated at room temperature away from light for 15 min. Annexin V and PI staining were analyzed using a BD FACSAria II flow cytometer (BD Biosciences).

### Tumor xenografts

Lentiviral vectors (pGLVH 1/GFP+Puro) targeting human RAB5A were generated by Shanghai GenePharma Co., Ltd. (Shanghai, China). MDA-MB-231 cells were cultured until they reached 80–90% confluence and were then inoculated in six-well plates. Cells were washed twice, trypsin-digested, and subsequently transferred to 2 mL DMEM supplemented with 10% FBS. Cells at a density of 1.5 × 10^6^ cells/well were mixed with lentiviral vectors and incubated at 37°C and 5% CO_2_ for 24 h. Cells were then transferred to a 6 cm petri dish and cultured until 80–90% confluence. Subsequently, 0.1 μg/mL puromycin was added for selection of stably transfected cells. The medium was refreshed every day, and puromycin selection was performed for four continuous days. The remaining cells were considered stably transfected cells, and western blotting was conducted for verification. The cells were cultured for another 2 weeks before further use.

All animal experiments were reviewed and approved by the Ethics Committee of the Affiliated Tumor Hospital Xinjiang Medical University (G-2020051). For the tumor growth assay, 6–8-week-old nude mice were divided into four randomized groups, and MDA-MB-231 cells alone (5 × 10^5^) or in combination with THP-1 cells (5 × 10^5^) were subcutaneously injected into the flank of each mouse. After 7 days, we began measuring the tumor size every 7 days using digital Vernier calipers. Twenty-eight days after cell injection, the mice were sacrificed to collect the tumors and visually examine them. According to the Ethics Committee Guidelines, the maximal tumor volume in animals in this study did not exceed 2000 mm^3^ or 10% of body weight.

### Immunohistochemistry

The tumor xenografts were fixed with 4% paraformaldehyde for 24 h, dehydrated with a graded series of ethanol solutions, and embedded in paraffin. The tissues were then sectioned into 4 μm slices. Subsequently, the sections were deparaffinized and rehydrated. After immersion in 3% H_2_O_2_, endogenous peroxidase activity was blocked. Slices were then blocked with 5% goat serum for 30 min. A primary antibody against CD163 (1:200, ab182422, Abcam) or CD68 (1:100, ab283654, Abcam) was added to the slices and incubated at 4°C overnight. The slices were washed with PBS and subsequently incubated with biotinylated secondary antibodies at 37°C for 1 h. Upon DAB staining, the slices were counterstained with hematoxylin for 5–10 min. The staining was examined under a light microscope. The CD163 and CD68 stainings were quantified using ImageJ software (NIH) with the IHC Profiler ImageJ plugin (Varghese *et al.* 2014).

### Statistical analysis

Data are expressed as mean ± s.d. or median with interquartile range. The normal distribution was first determined by the Kolmogorov–Smirnov test. The differences between two groups were evaluated by the Mann–Whitney *U* test or Student’s *t* test, and differences among three or more groups were tested by one-way ANOVA followed by Tukey’s *post hoc* test. *P* < 0.05 was considered to indicate statistical significance.

## Results

### RAB5A knockdown inhibits the secretion of exosomes in TNBC cells

**Figure 1:**
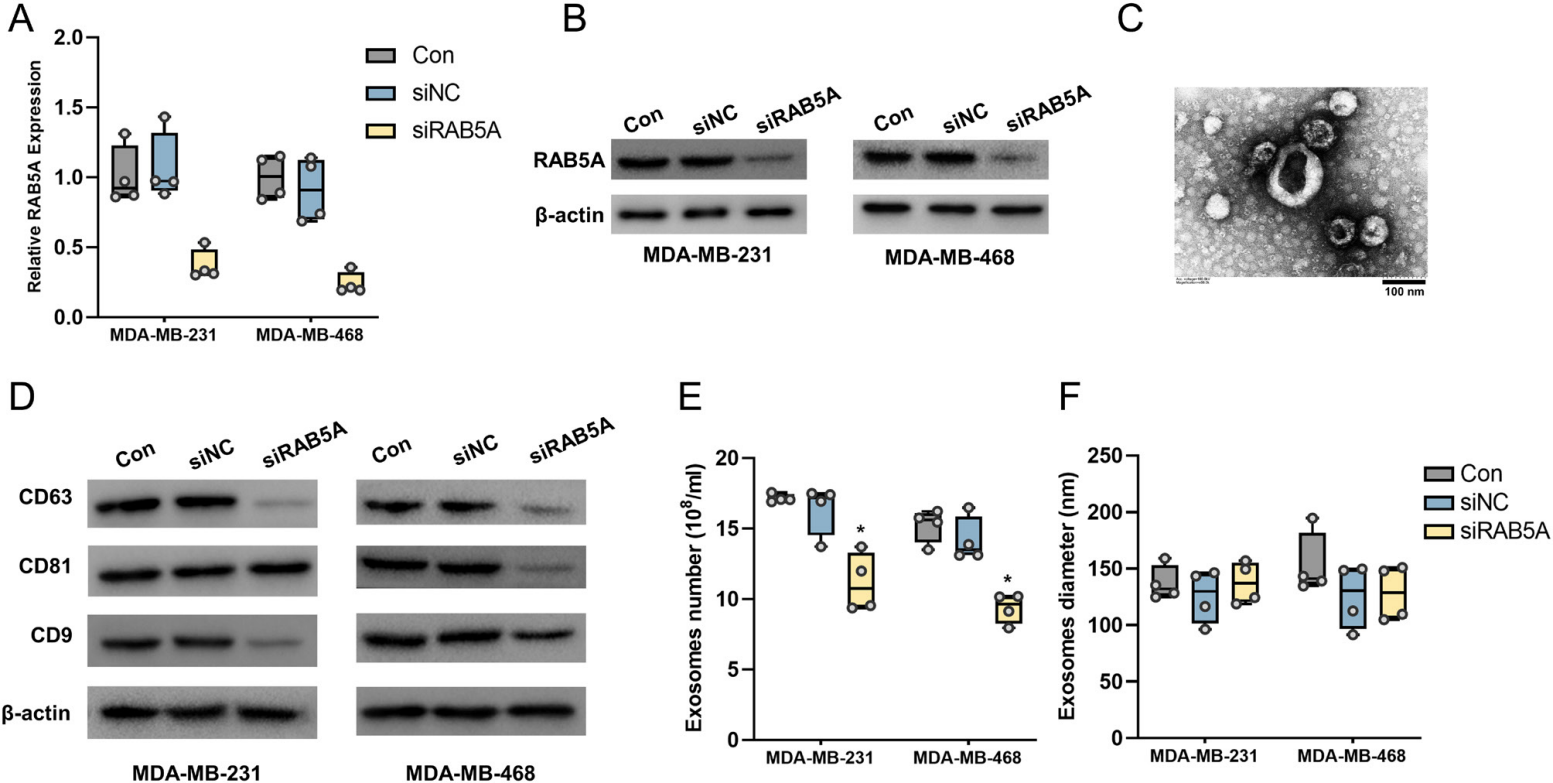
RAB5A knockdown in TNBC cells suppresses the secretion of exosomes. The mRNA (A) and protein (B) levels of RAB5A in MDA-MB-231 and MDA-MB-468 cells after transfection with RAB5A-targeting small interfering RNA. (C) Transmission electron microscopy image of exosomes from MDA-MB-231 cells, scale bar: 100 nm. (D) The protein levels of exosome hallmarks in the culture medium of MDA-MB-231 and MDA-MB-468 cells transfected with RAB5A siRNA. The number (E) and diameter (F) of exosomes isolated from MDA-MB-231 and MDA-MB-468 cells with RAB5A knockdown. *P < 0.05.

RAB5A knockdown TNBC cells were established by transfection of siRNAs targeting RAB5A, and our mRNA and protein results suggested lower expression of RAB5A in cells transfected with targeted siRNAs (Fig. 1A and B). Our previous study demonstrated the involvement of RAB5A in exosome secretion in MDA-MB-231 cells (Qiao *et al.* 2023). In the present study, two TNBC cell lines, MDA-MB-231 and MDA-MB-468, were employed for further investigation. The exosomes were first observed under a TEM. As indicated in Fig. 1C, the exosomes showed a classical cup-shape flattened sphere, with a diameter of ~100 nm (the largest possibly reaches up to 150 nm or over). Despite the definition of the exosome varies in diameter (Kalluri 2016, Steinbichler *et al.* 2017, Zhu *et al.* 2020), it is rational to suppose that the vesicles isolated in this study are exosomes. Exosome hallmarks, including CD63, CD81, and CD9, were tested in cell culture medium. RAB5A-depleted cells exhibited apparent decreases in CD63 and CD9 in both TNBC cell lines, whereas for CD9, a decrease was solely observed in MDA-MB-468 cells (Fig. 1D). Our NTA results also indicated that the exosome number in the culture medium of RAB5A-knockdown cells was less than that in the culture medium of control or siNC-transfected cells (Fig. 1E), whereas the diameter of exosomes was not varied among the three groups (Fig. 1F). These results indicated that knockdown of RAB5A in TNBC cells suppressed the secretion of exosomes.

### Involvement of RAB5A in TNBC-derived exosome-mediated polarization of macrophages

**Figure 2:**
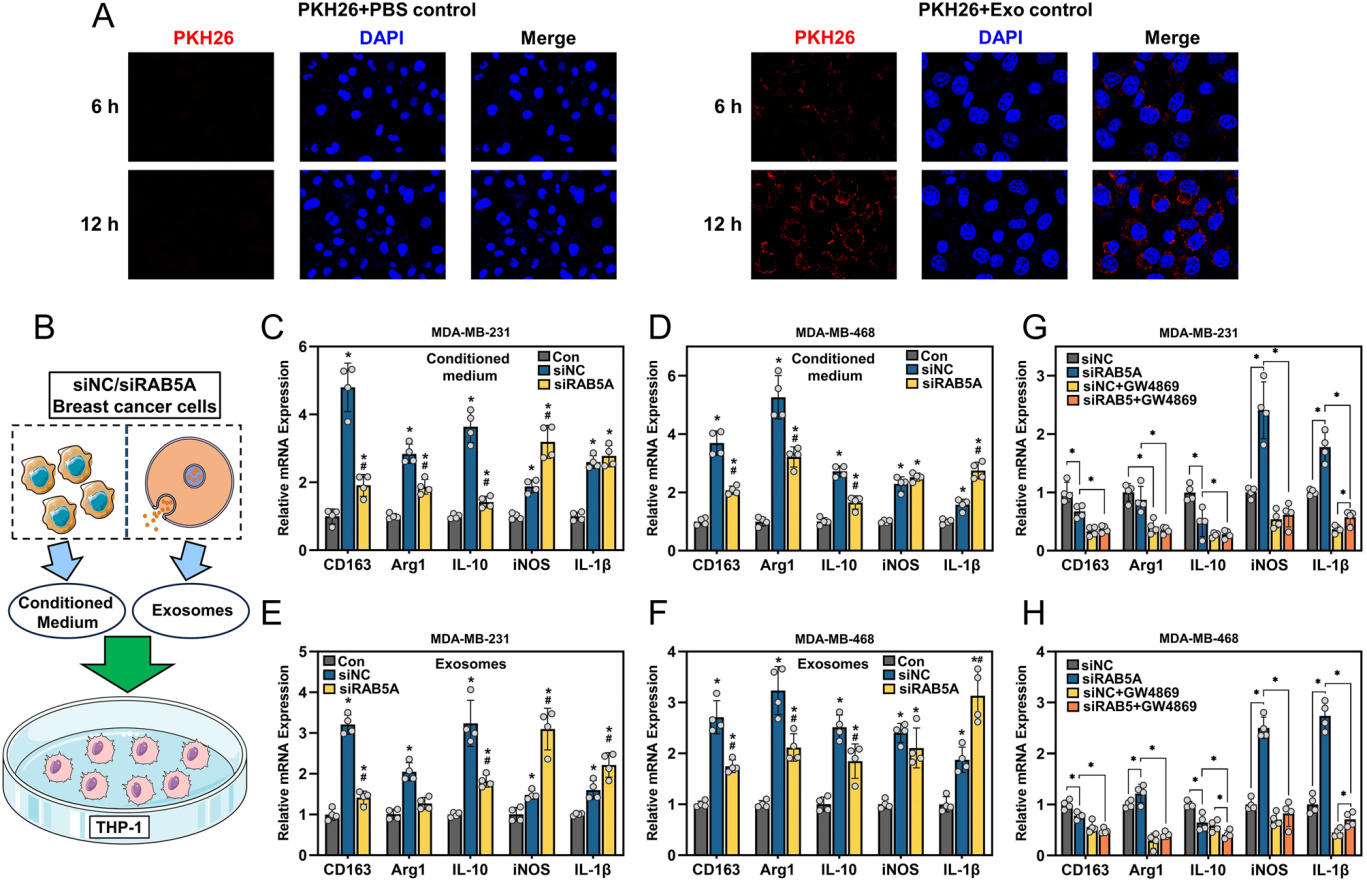
RAB5A knockdown in TNBC cells alters macrophage polarization. (A) Internalization of PKH26-labeled exosomes by THP-1 cells at 6 and 12 h, PKH26 plus PBS was served as the negative control. (B) The schematic diagram of the experiment design: two TNBC cells were cultured for 48 h, and the cultured medium was collected in duplicates, one is used to directly incubated with THP-1 cells, the other was used for exosome isolation. The isolated exosomes were first quantified, and diluted to equal concentrations, and then added to the THP-1 cells. The expression of M2 (CD163, Arg1 and IL-10) and M1 (iNOS and IL-1β) markers in PMA-pretreated THP-1 cells after incubation with conditioned medium from MDA-MB-231 (C) and MDA-MB-468 (D) cells with RAB5A knockdown, *P < 0.05, vs Con, ^#^ *P* < 0.05, vs siNC. The expression of M2 and M1 markers in THP-1 cells incubated with exosomes isolated from MDA-MB-231 (E) and MDA-MB-468 (F) cells with RAB5A knockdown, *P < 0.05, vs Con, ^#^ *P* < 0.05, vs siNC. The expression of M2 and M1 markers in THP-1 cells treated with conditioned medium from MDA-MB-231 (G) and MDA-MB-468 (H) cells, which were pretreated with an exosome biogenesis and release inhibitor, GW4869, *P < 0.05.

It is well documented that tumor-derived exosomes are widely engaged in the regulation of the tumor microenvironment, especially the polarization of macrophages (Baig *et al.* 2020). First, the internalization of TNBC-derived exosomes by THP-1 cells was evaluated by PKH26 staining. The results in Fig. 2A demonstrated that PKH26-labeled exosomes were internalized by THP-1 cells at 6 h of incubation, and at 12 h, more PKH26 signal was observed around the nuclei of THP-1 cells. To investigate the impact of tumor cell-derived exosomes on the polarization of macrophages, we incubated the THP-1 cells with conditioned medium or isolated exosomes (Fig. 2B). THP-1 cells (PMA-pretreated) were cultured with conditioned medium harvested from two TNBC cell lines, as indicated in Fig. 2C and D. Both M1 and M2 markers of THP-1 cells were largely induced by incubation with conditioned medium from siNC and siRAB5A TNBC cells compared to the control (THP-1 cells alone, PMA-pretreated). Additionally, similar results were observed when THP-1 cells were incubated with TNBC cell-derived exosomes (Fig. 2E and F). We further used GW4869, an exosome biogenesis inhibitor, to pretreat the TNBC cells and collected the conditioned medium to incubate with the THP-1 cells. The results in Fig. 2G and H indicated that by blocking exosome biogenesis in TNBC cells, the capability of conditioned medium to induce the polarization of macrophages was largely impaired.

### RAB5A-mediated exosomal miR-21 secretion

**Figure 3:**
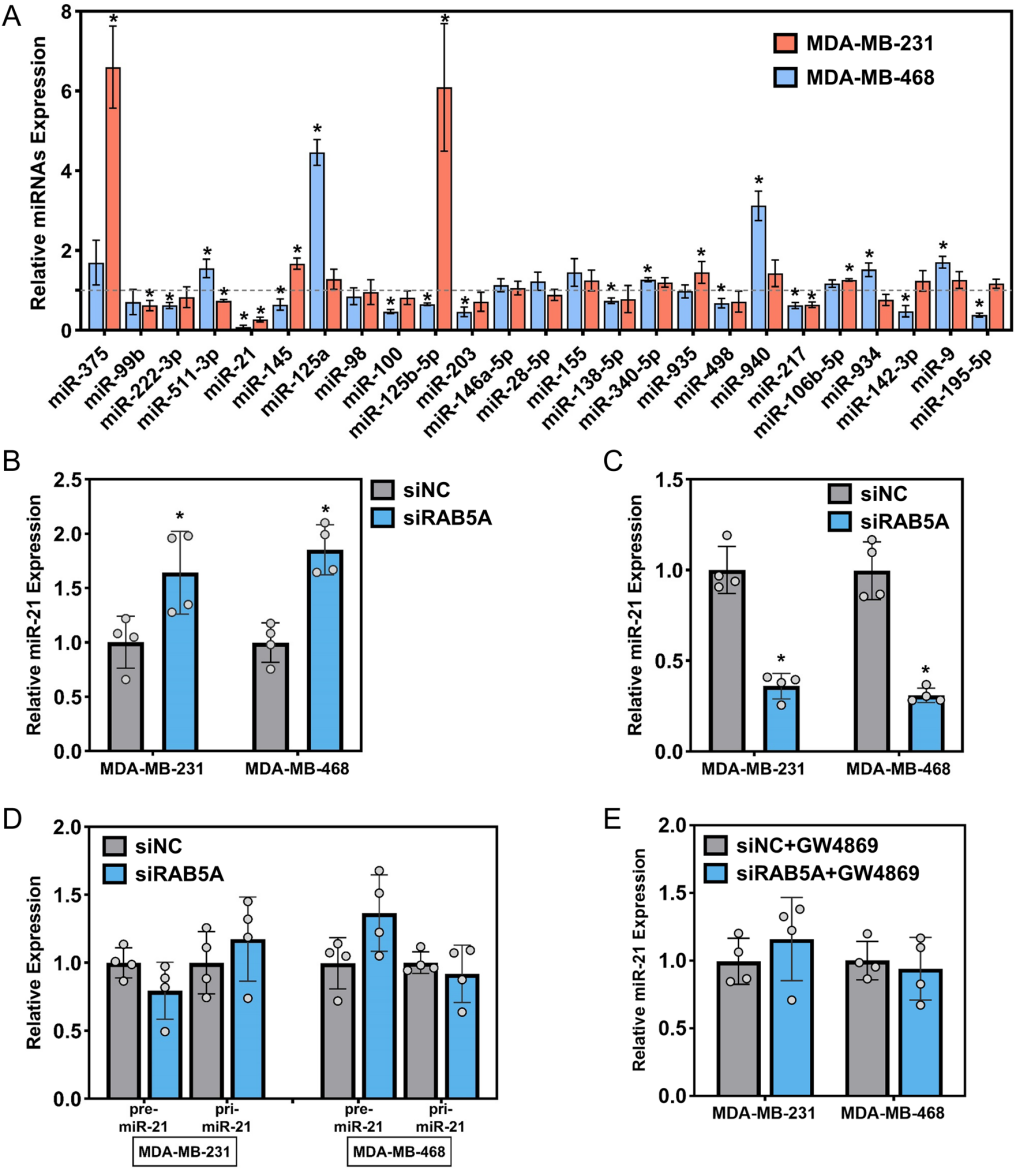
RAB5A knockdown in TNBC cells inhibits the release of miR-21. (A) The expression of macrophage polarization-associated miRNAs in exosomes from two TNBC cell lines. miRNA expression was normalized to that of siNC, *P < 0.05, siRAB5A vs siNC. Intracellular (B) and culture medium-released (C) miR-21 expression in two TNBC cell lines after RAB5A knockdown. (D) The intracellular expression of primary miR-21 (pri-miR-21) and precursor miR-21 (premiR-21) in two TNBC cell lines with RAB5A depletion. Intracellular mature miR-21 level in two TNBC cell lines with RAB5A knockdown after GW4869 administration. *P < 0.05, vs siNC.

To investigate whether RAB5A regulates the secretion of exosomal miRNAs, a series of putative miRNAs previously documented which were involved in macrophage polarization (Supplementary Table 2) were selected and tested in isolated exosomes. As indicated in Fig. 3A, the expression of miR-21 in two TNBC cell lines was consistently decreased after RAB5A knockdown. Given the decreased expression of miR-21 in exosomes, we further tested its expression in cells and culture medium. Intriguingly, ectopic expression of intracellular miR-21 was observed after RAB5A depletion in both cell lines (Fig. 3B). However, the expression of miR-21 in the culture medium of the two cell lines was decreased (Fig. 3C). To examine where RAB5A knockdown alters the biogenesis or secretion of miRNA, we subsequently determined the intracellular level of primary miR-21 (pri-miR-21) and precursor miR-21 (premiR-21), and our results suggested that neither pri-miR-21 nor premiR-21 was unaltered in RAB5A knockdown cells (Fig. 3D). Furthermore, GW4869 was applied to block the biogenesis and secretion of exosomes in TNBC cells, and a parallel level of miR-21 was investigated in two cell lines with or without RAB5A knockdown (Fig. 3E). These results implied the engagement of RAB5A in the secretion of miR-21.

### Exosomal miR-21 regulates the polarization of macrophages

**Figure 4:**
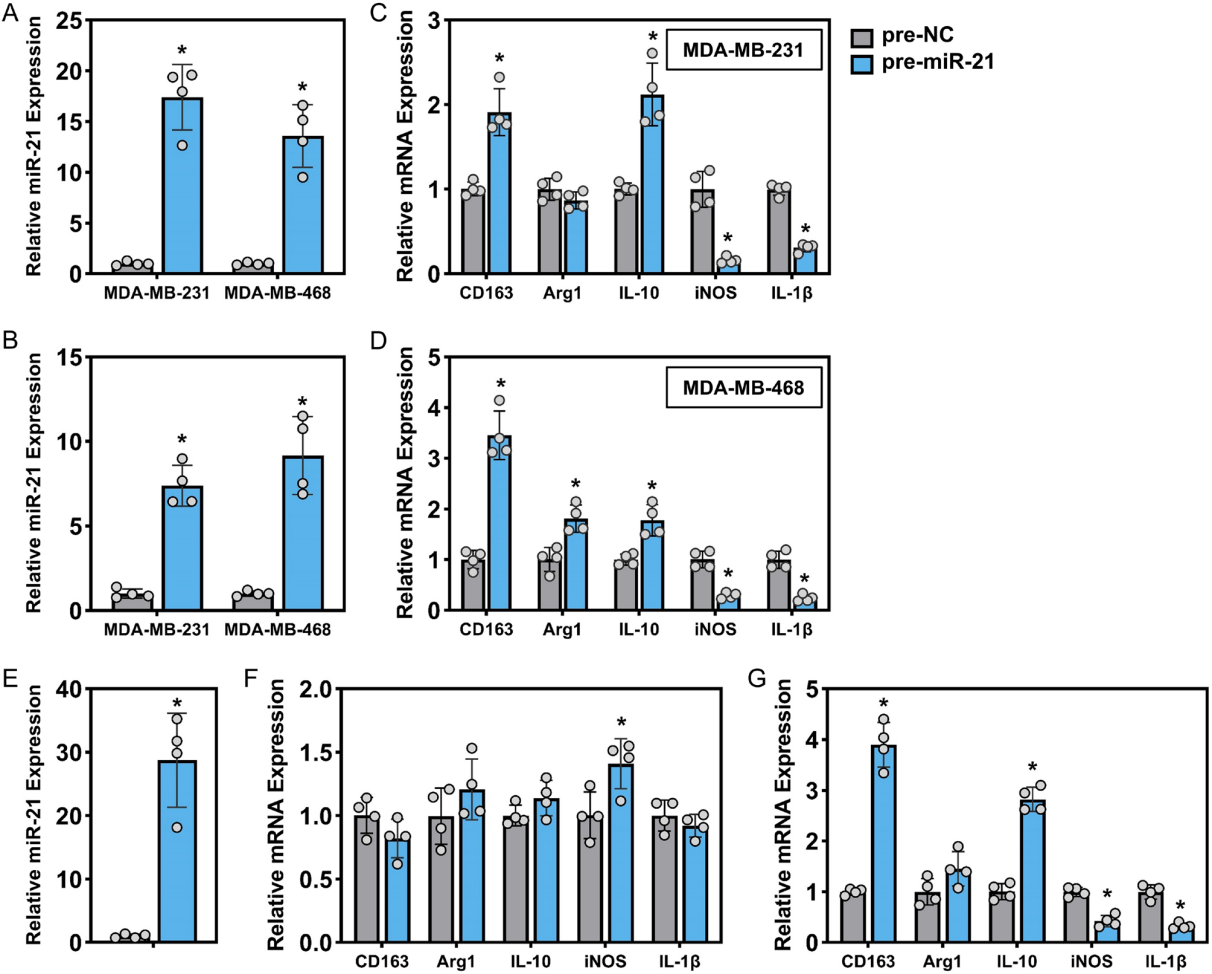
Exosomal miR-21 regulates the polarization of macrophages. Intracellular (A) and exosomal (B) miR-21 levels in two TNBC cell lines transfected with pre-NC or premiR-21. The expression of M2 and M1 markers in THP-1 cells treated with exosomes extracted from MDA-MB-231 (C) and MDA-MB-468 (D) cells, two TNBC cell lines, were transfected with pre-NC or premiR-21. (E) The expression of miR-21 in THP-1 cells transfected with premiR-21. (F) The expression of M2 and M1 markers in THP-1 cells after miR-21 overexpression. THP-1 cells were solely pretreated with PMA with no other treatments. (G) The expression of M2 and M1 markers in THP-1 cells after miR-21 overexpression. THP-1 cells were pretreated with PMA and subsequently incubated with conditioned medium from parental MDA-MB-231 cells. *P < 0.05, vs pre-NC.

In light of the results mentioned above, we constructed miR-21-overexpressing TNBC cells to further investigate the role of miR-21 in regulating macrophage polarization. The expression of miR-21 was dramatically elevated by transfection of premiR-21 in TNBC cells and exosomes (Fig. 4A and B). We first isolated exosomes from TNBC cells and incubated them with THP-1 cells pretreated with PMA. The expression of M1 and M2 markers was examined in THP-1 cells. In cells treated with exosomes derived from miR-21-overexpressing TNBC cells, the expression of IL-10 and CD163 was largely induced, along with the inhibited expression of iNOS and IL-1β (Fig. 4C and D). Additionally, the overexpression of miR-21 was conducted in THP-1 cells by premiR-21 transfection (Fig. 4E), and the results in Fig. 4F suggested that forced miR-21 expression in macrophages failed to induce polarization, as assessed by the unaltered expression of M1/M2 markers. However, when THP-1 cells with miR-21 overexpression were treated with conditioned medium from TNBC cells (MDA-MB-231), the M2 markers CD163 and IL-10 were upregulated, but Arg1 was not, whereas the expression of the M1 markers iNOS and IL-1β was significantly suppressed (Fig. 4G).

### PELI1 is identified as the target of miR-21

**Figure 5:**
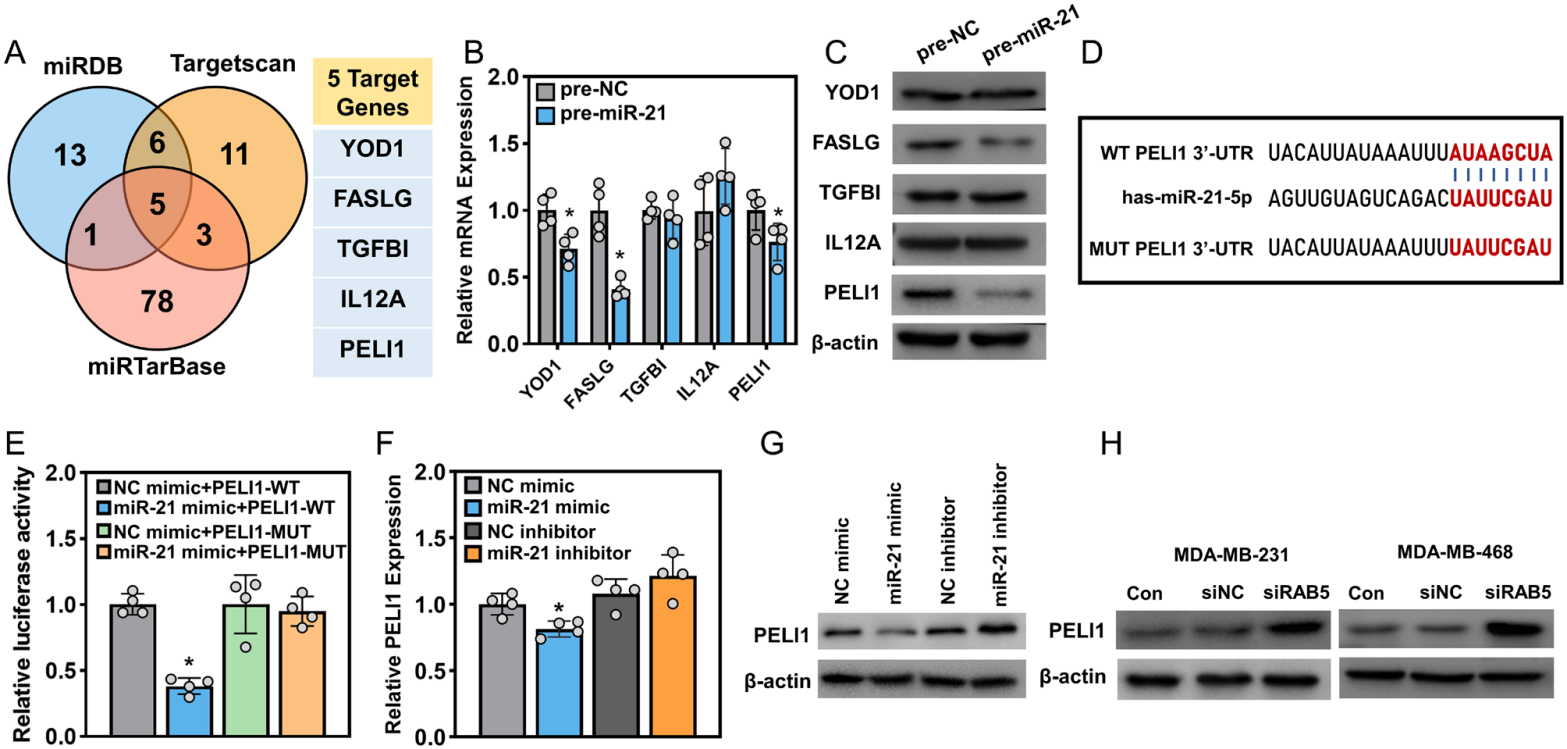
miR-21 targets the expression of Pellino-1 (PELI1) in THP-1 cells. (A) Venn diagram of targets of miR-21 predicted in three online databases, miRTarBase, miRDB, and TargetScan. The mRNA (B) and protein (C) expression levels of five candidate target genes of miR-21, *P < 0.05, vs pre-NC. (D) The wild-type (WT) and mutant (MUT) binding site of the PELI1 3′-untranslated region (3′-UTR) with miR-21. (E) The luciferase activity of PELI1-WT and MUT in THP-1 cells treated with NC mimic or miR-21 mimic, *P < 0.05, vs NC mimic+PELI1-WT. The mRNA (F) and protein (G) levels of PELI1 in THP-1 cells treated with NC mimic or miR-21 mimic, *P < 0.05, vs NC mimic. (H) The protein expression of PELI1 in THP-1 cells after incubation with conditioned medium from TNBC cells with RAB5A knockdown.

miRNAs, defined as 20–22 nt RNA molecules, negatively regulate gene expression in eukaryotic organisms, and their role in tumorigenesis has been intensively studied in the last two decades (McManus 2003, Croce & Calin 2005). To ascertain the targeted gene(s) in miR-21-mediated macrophage polarization, we predicted the miR-21 targets using three widely used web tools, including miRTarBase (Huang *et al.* 2020), miRDB (Chen & Wang 2020), and TargetScan (Agarwal *et al.* 2015). The top 25 ranked results obtained from miRDB and TargetScan were filtered. For miRTarBase, the results with at least one ‘strong evidence’ and two ‘weak evidence’ were selected. The results were evaluated as a Venn plot, and these three databases shared five targets (Fig. 5A). We tested the expression of these five target genes in THP-1 cells overexpressing miR-21 at both the mRNA and protein levels. YOD1, FASLG, and PELI1 were decreased at the mRNA level in the presence of premiR-21, while the largest alteration was identified in the protein abundance of PELI1 (Fig. 5B and C); thus, we next focused on PELI1 to reveal its interaction with miR-21. The binding site between miR-21 and the PELI1 mRNA sequence was presented, and a mutant in the PELI1 3′-UTR was introduced (Fig. 5D). THP-1 cells were transfected with NC or miR-21 mimic, along with luciferase vectors with wild-type or mutated binding sites of the PELI1 3′-UTR, and the luciferase activity was subsequently examined. As indicated in Fig. 5E, transfection with wild-type binding sites led to a decrease in luciferase activity in the presence of miR-21 mimic, while for other transfections, the luciferase activity remained unchanged when compared to the control (NC mimic+pGL3-PELI1-WT). Furthermore, the mRNA and protein levels of PELI1 were detected in THP-1 cells transfected with miR-21 mimic or miR-21 inhibitor. Notably, the miR-21 mimic levels slightly decreased, whereas the miR-21 inhibitor failed to impact the mRNA level of PELI1 in THP-1 cells (Fig. 5F). More apparent changes were observed at the protein level; cells transfected with miR-21 mimics showed a reduction in PELI1 protein by almost two-thirds, while cells transfected with miR-21 inhibitors showed a 1.7-fold increase in PELI1 protein levels (Fig. 5G). We next tested whether the administration of tumor cell-derived exosomes influences the expression of PELI1 in macrophages. THP-1 cells (PMA-pretreated) were incubated with exosomes isolated from two TNBC cell lines with or without RAB5A knockdown, and THP-1 cells treated with exosomes from the parent TNBC cells were used as the control. The PELI1 protein in THP-1 cells was elevated after treatment with exosomes from RAB5A-depleted TNBC cells (Fig. 5H). The above results suggested that PELI1 is the target of miR-21 and that RAB5A in tumor cells has the capability to regulate the expression of PELI1 in macrophages in an exosomal miR-21-dependent manner.

### miR-21 regulates the polarization of macrophages via PELI1

**Figure 6:**
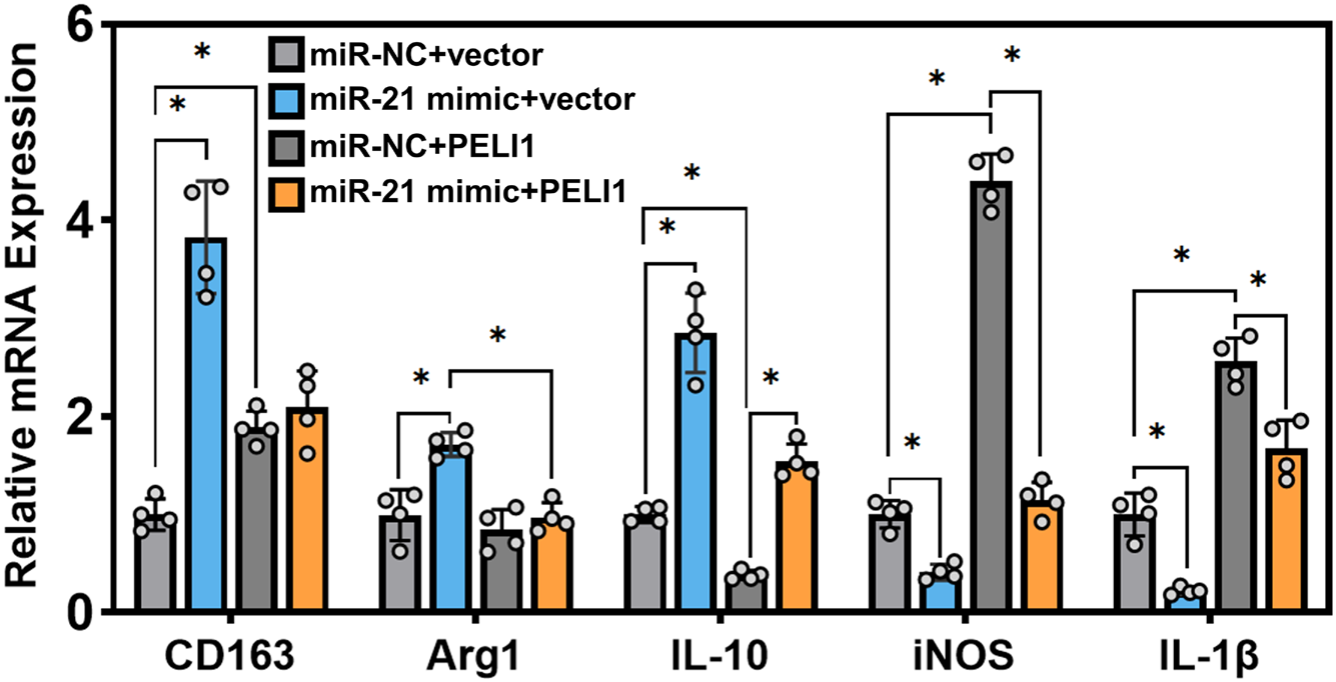
miR-21 overexpression potentiates the M2 polarization of macrophages. The expression of M2 and M1 markers in THP-1 cells (PMA-pretreated) overexpressing miR-21 or PELI1 alone or in combination with the incubation of conditioned medium from MDA-MB-231cells, *P < 0.05.

Given that RAB5A modulates the polarization of macrophages by secreting miR-21-harbored exosomes, we were motivated to explore whether miR-21-mediated macrophage polarization is PELI1 dependent. The overexpression of miR-21 or PELI1 was carried out in THP-1 cells alone or in combination, followed by culture with conditioned medium from MDA-MB-231 cells. The results in Fig. 6 suggested that miR-21-overexpressing THP-1 cells exhibited increased expression of M2 markers and decreased expression of M1 markers, which was partially abrogated by PELI1 overexpression.

### Implication of macrophages in the malignancy of TNBC cells

**Figure 7:**
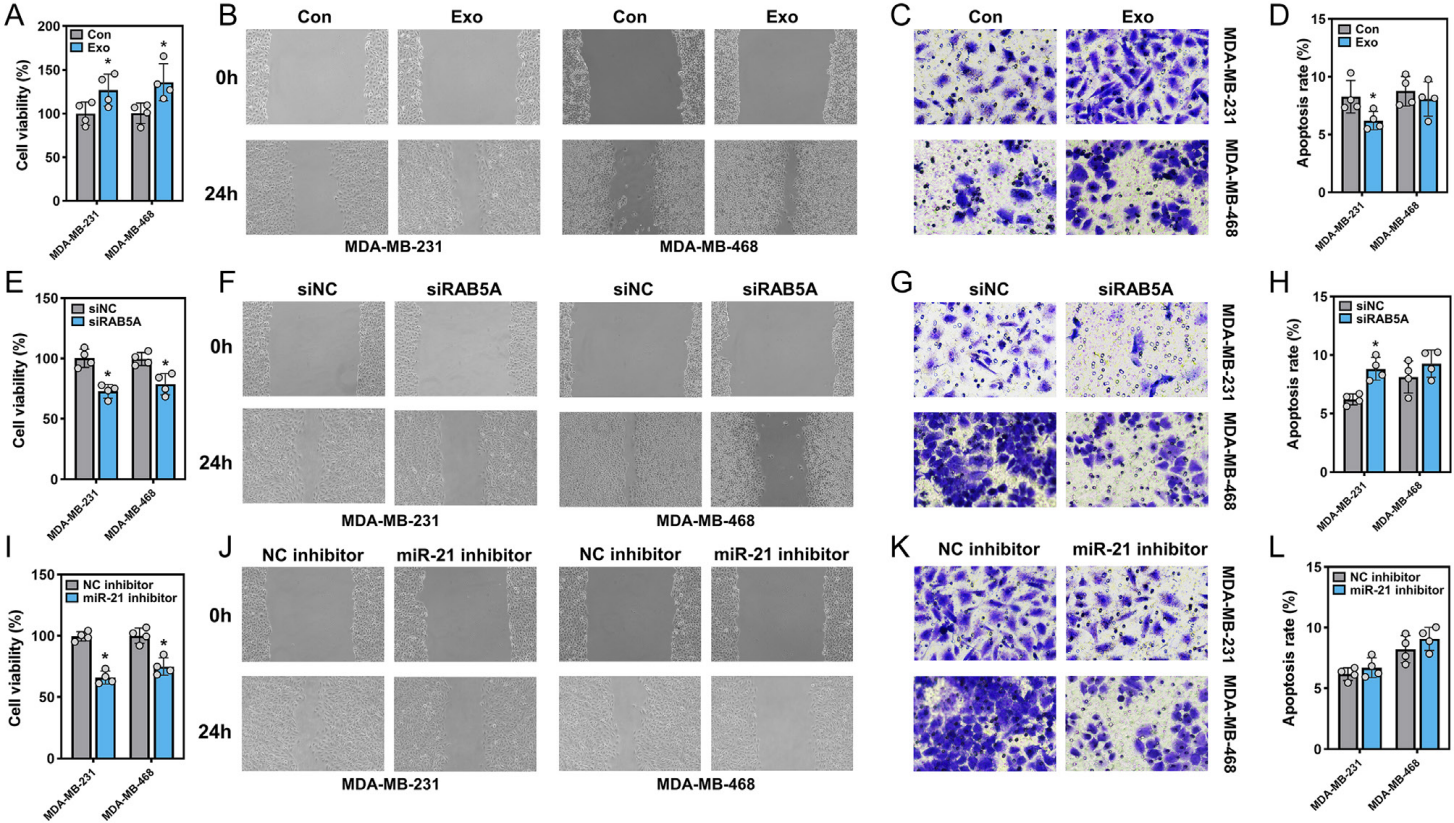
Macrophages induced malignance of TNBC cells. Cell viability (A), migration (B), invasion (C), and apoptosis (D) of two TNBC cell lines. PMA-pretreated THP-1 cells were first treated with or without exosomes from TNBC cells, and the conditioned medium from THP-1 cells was subsequently incubated with TNBC cells, *P < 0.05, vs Con. Cell viability (E), migration (F), invasion (G), and apoptosis (H) of two TNBC cell lines. PMA-pretreated THP-1 cells were first treated with exosomes derived from TNBC cells with or without RAB5A knockdown, and the conditioned medium from THP-1 cells was subsequently incubated with TNBC cells, *P < 0.05, vs siNC. Cell viability (I), migration (J), invasion (K), and apoptosis (L) of two TNBC cell lines. THP-1 cells were transfected with NC or miR-21 inhibitors. After PMA pretreatment, THP-1 cells were cocultured with TNBC cells in a Transwell coculture system, *P < 0.05, vs NC inhibitor.

The abovementioned results, to some extent, explained the role of TNBC in shaping macrophages, in which RAB5A and exosomal miR-21 were involved. The reciprocal interactions between tumor cells and macrophages have been widely documented (Anderson & Simon 2020). Tumor cells themselves have the capability to create a microenvironment to facilitate their development, and macrophages are an essential element, specifically tumor-associated macrophages (TAMs). In this study, TAMs were generated by treatment with tumor-derived exosomes, while those without treatment were employed as the control. We incubated the conditioned medium from TAMs with two TNBC cell lines, and their cellular behaviors were further inspected. Significantly, both MDA-MB-231 and MDA-MB-468 cells treated with conditioned medium from TAMs exhibited increased viability (Fig. 7A) compared to their counterparts treated with control macrophages. Additionally, these TAM-stimulated TNBC cells gained advantages in migration and invasion, as assessed by wound-healing and Transwell assays, respectively (Fig. 7B and C). However, the cell apoptosis in the two TNBC cell lines was not consistent, and a decrease in the apoptosis rate was observed only in MDA-MB-231 cells (Fig. 7D). We next asked whether macrophages stimulated with exosomes from RAB5A-silenced TNBC cells still had the potential to induce transformation. Macrophages were incubated with exosomes derived from TNBC cells transfected with siNC or siRAB5A, and subsequently, the macrophage-cultured medium was used for TNBC cell cultivation (these TNBC cells had received no treatments). Apparently, when cultured with conditioned medium from macrophages incubated with RAB5A-depleted tumor exosomes, TNBC cell viability was suppressed (Fig. 7E), as were the migration and invasion capabilities (Fig. 7F and G). While the situations in cell apoptosis seemed inconsistent, the apoptosis rate in MDA-MB-231 cells was slightly induced by treatment with conditioned medium from the ‘siRAB5A’ group macrophages when compared to the control, whereas it remained unchanged for MDA-MB-468 cells between the two groups (Fig. 7H).

A coculture system of TNBC cells and miR-21-inhibited macrophages was established to determine macrophage-mediated tumor cell malignancy in another way. miR-21 inhibitor-transfected macrophages were seeded on the upper layer of a Transwell chamber, and TNBC cells were seeded in the lower chamber. After incubation, the viability, migration, and invasion of TNBC cells were determined. As anticipated, miR-21-blocked macrophages exerted a tumoricidal effect, or to be more precise, miR-21-blocked macrophages could not facilitate proliferation, migration, and invasion as well as control-transfected macrophages (Fig. 7I, J, K, and L).

### RAB5A knockdown suppressed tumor growth *in vivo*

**Figure 8:**
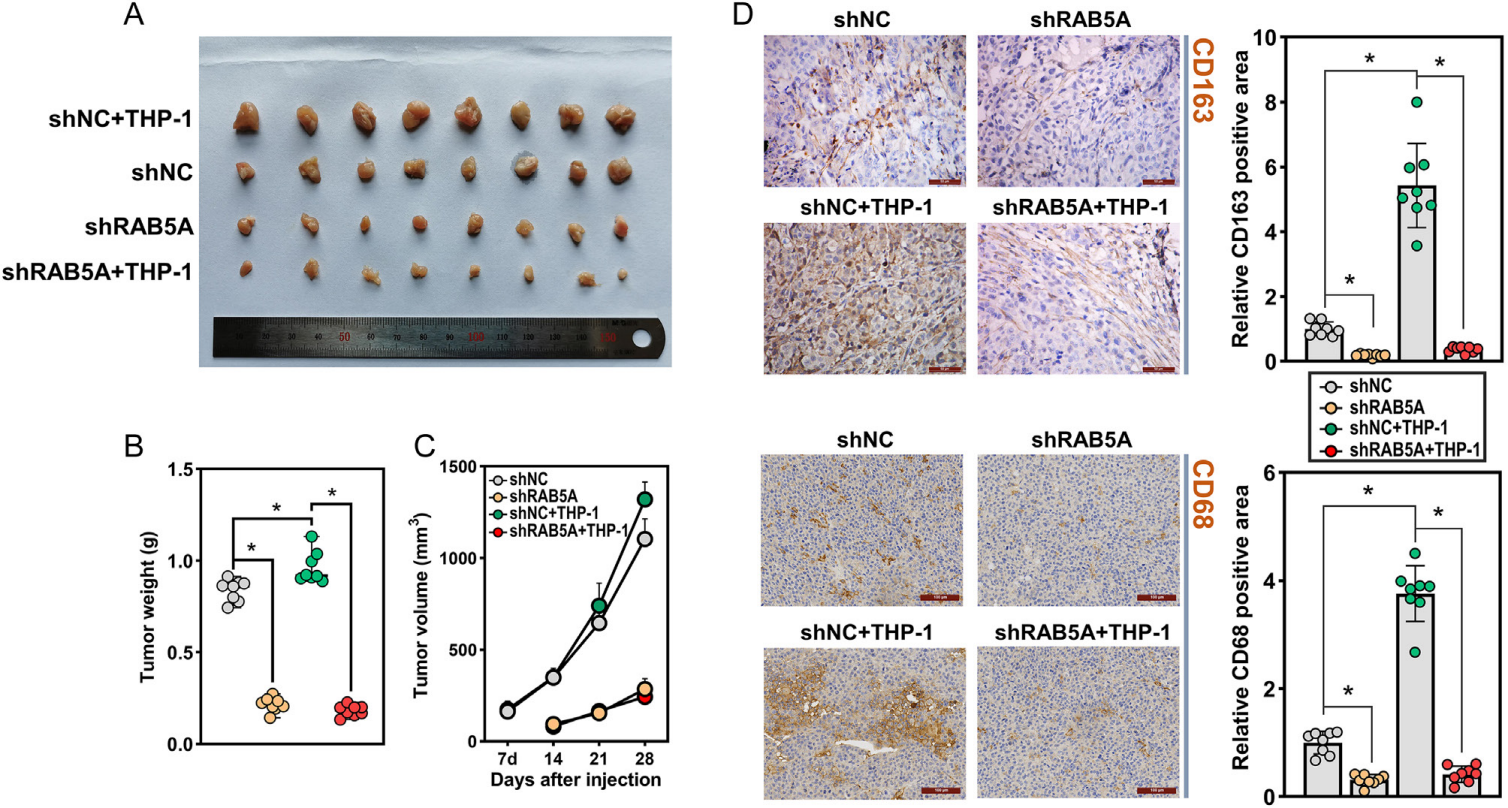
RAB5A knockdown suppressed tumor growth *in vivo*. PMA-pretreated THP-1 cells and MDA-MB-231 cells (lentivirus-mediated RAB5A knockdown) were coinjected into the flank of female nude mice. (A) Pictures of subcutaneous tumors. Weight (B) and growth curve (C) of tumors generated in mice, *P < 0.05. (D) Immunohistochemical staining of CD163 and CD68 in mouse xenografts with their corresponding quantification results, *P < 0.05.

The interactions between TNBC cells and TAMs were further investigated *in vivo* through a nude mouse xenograft model. TAMs were generated by coculture with TNBC cells. MDA-MB-231 cells (5 × 10^5^) with or without RAB5A knockdown, alone or in combination with TAMs (5 × 10^5^), were subcutaneously injected into the flanks of female nude mice. The mice injected with RAB5A-depleted MDA-MB-231 cells developed smaller tumors than those injected with control TNBC cells (Fig. 8A and B). In addition, the tumors in mice coinjected with shNC-MDA-MB-231 cells and TAMs were larger than those in mice that received TNBC cells alone. Intriguingly, tumor volume in mice injected with RAB5A-depleted MDA-MB-231 cells and THP-1 cells seem not to differ from those with only RAB5A-depleted MDA-MB-231 cell injection (Fig. 8C). By detecting the M2 macrophage markers CD163 and CD68, we noticed that tumors with shNC TNBC cell and THP-1 cell injections exhibited higher CD163 and CD68 staining, while the least CD163- and CD68-positive staining was observed in those with shRAB5A TNBC cell and THP-1 cell injections (Fig. 8D). These results indicated that RAB5A knockdown in TNBC cells has the potential to suppress tumor growth *in vivo*, and this effect might be partially mediated by macrophage polarization.

## Discussion

The cross talk between tumor cells and their microenvironment is attracting increasing attention and is believed to be a promising therapeutic target (Bejarano *et al.* 2021). Tumor cells have the capability to remodel their surroundings to support their development, and the tumor microenvironment, in turn, potentiates the development and metastasis of the tumor (Baghban *et al.* 2020). Macrophages are a critical component of the tumor microenvironment, and their reciprocal interactions with tumor cells have been well documented. In our previous study, we focused on RAB5A and its role in the development of breast cancer, especially TNBC. We identified RAB5A as an oncogene in TNBC, and its potential effects on macrophage polarization were preliminarily investigated (Qiao *et al.* 2023). The molecular basis of RAB5A in TNBC progression was further deepened in the current study. RAB5A was implicated in exosome secretion and the polarization of macrophages. Furthermore, miR-21 was identified as the most impacted exosomal miRNA upon RAB5A depletion. By scanning the potential targets of miR-21, PELI-1 was subsequently proposed as a candidate and further verified. By knockdown of RAB5A, TNBC cells release fewer exosomal miR-21, which is taken up by surrounding macrophages. Upon entry into macrophages, miR-21 can suppress the production of PELI1, and less PELI1 in macrophages in the presence of tumor cells or tumor cell-derived stimuli hinders the development of an M1-like phenotype. Additionally, such a change in macrophages will in turn affect the fate of tumor cells.

PELI1 is an E3 ubiquitin ligase belonging to the Pellino family, and its implication in regulating TLR signaling has been widely reported (Wang *et al.* 2017). Recent investigations have noted that PELI1 plays a crucial role in mediating the polarization of the macrophages. For instance, Kim and colleagues demonstrated that PELI1 promotes the K63-linked ubiquitination of IRF5 and thus potentiates the M1 polarization in macrophages (Kim *et al.* 2017). Furthermore, the authors also observed that the presence of PELI1 suppresses the M2c polarization of macrophages and exhibits a tumoricidal effect (Kim *et al.* 2019). Moreover, investigations by independent research groups have shown that the deficiency of PELI1 in macrophages impairs their transformation toward the M1 pro-inflammatory phenotype (Chen *et al.* 2023, Mo *et al.* 2023). In addition, a similar impact of PELI1 is also observed in microglia, as PELI1 depletion inhibits M1 but facilitates M2 polarization (Wang *et al.* 2020). Collectively, the previous findings were consistent with our results, suggesting the key role of PELI1 in macrophage polarization.

The Rab5 protein has been recognized to play a role in endosomal maturation, exosome biogenesis, and transportation (Hurley & Odorizzi 2012, Chen *et al.* 2021). Additionally, investigations by Gorji-bahri suggest the potential involvement of RAB5A in exosome secretion during cancer progression (Gorji-Bahri *et al.* 2021*a*, *b*). The role of exosomes in intercellular communication, especially during tumor progression, has been extensively studied. Exosomes deliver bioactive molecules, such as proteins, mRNA, and miRNA, to cells in the tumor microenvironment to regulate immunity (Nabariya *et al.* 2020). The role of exosomes in tumor immunity is alterable, with both immunostimulatory and immunosuppressive effects, mainly through the regulation of relevant immune cells in the tumor microenvironment, including dendritic cells, regulatory T cells, and tumor-associated macrophages (Sheybani *et al.* 2020). miRNAs are important exosomal components and are widely found to be involved in cancer development and progression (Nabariya *et al.* 2020). miRNAs can act as inhibitors or promoters to regulate some key proteins and signaling pathways to determine cancer progression, such as cell proliferation, apoptosis, metastasis, and invasion (Bhagirath *et al.* 2018). In the tumor microenvironment, exosomal miRNAs can affect the immune system in different ways, such as regulating macrophage activation and reprogramming immunoreactive factors. Tumor-associated macrophages (TAMs) are resident or recruited macrophages in the tumor microenvironment, where they are key components (Chen *et al.* 2019). Numerous studies have noted that TAMs can effectively promote tumor progression and metastasis (Yu *et al.* 2021). Activated macrophages are usually classified into M1 (proinflammatory) and M2 (anti-inflammatory) phenotypes, viz. macrophage polarization, depending on the external stimulus. Typically, M1 macrophages promote inflammation by releasing or promoting the release of inflammatory factors, whereas M2 macrophages tend to suppress the inflammatory response and facilitate tissue repair (Beltraminelli & De Palma 2020). We noticed that the depletion of RAB5A in tumor cells reduced the staining of M2-macrophage marker CD163, as well as the pan-macrophage marker CD68, in mouse xenograft, this alteration of both macrophage and M2-macrophage might be attributed to the less transformation of M1 to M2 macrophage and the reduced infiltration of macrophage, which deserved further investigation. Despite the well-established knowledge about macrophage polarization, to date, exosomal miRNA-driven macrophage polarization is still extremely attractive to scientists and clinicians, especially in antitumor therapy (Baig *et al.* 2020). It was previously documented that miR-21 promotes the polarization of M2 macrophages and the progression of various cancers (Hsieh *et al.* 2018, Xi *et al.* 2018, Ren *et al.* 2019). In our current study, miR-21 was identified as the executor in RAB5A-mediated macrophage polarization, and the loss-of-function of RAB5A in TNBC cells did not alter the expression of pri- or premiR-21 but slightly increased the accumulation of intracellular mature miR-21. Thus, we speculated that silencing RAB5A leads to an impairment of exosome biogenesis and delivery, subsequently causing a decrease in exosome secretion by tumor cells into the surroundings. Internalized miR-21 targets the expression of PELI1 in macrophages, mainly in a posttranscriptional regulatory manner, since the miR-21 mimic slightly decreased PELI1 mRNA but dramatically decreased PELI1 protein. In addition, the polarization of macrophages seems not to be induced by miR-21 alone but by other signaling derived from TNBC cells, as evidenced by the fact that miR-21 overexpression in THP-1 cells fails to induce polarization; a similar perspective was previously proposed by Xi and colleagues (Xi *et al.* 2018).

The interactions between tumor cells and macrophages are usually reciprocal, and although RAB5A-deficient TNBC cells still have the capability to influence the surrounding macrophages, the balance between M1 and M2 macrophages changes to develop a microenvironment that is not suitable for tumor cell survival, similar to normally expressed RAB5A. However, how polarized macrophages impact the development of tumor cells is less observed in our present study, which deserves further investigation.

Collectively, our study deepens the understanding of the role of RAB5A in tumor progression, proposes the potential molecular basis of RAB5A-mediated macrophage polarization in an exosomal miR-21-dependent manner, and helps to develop RAB5A- or exosome-based tumor therapeutic strategies.

## Supplementary Materials

Supplementary Material

## Declaration of interest

The authors declare that there is no conflict of interest that could be perceived as prejudicing the impartiality of the study reported.

## Funding

This work was supported by the Natural Science Foundation Project of Xinjiang Uygur Autonomous Region (No.: 2022D01C526).

## Ethical approval

Ethical approval was obtained from the Ethics Committee of Xinjiang Medical University affiliated Tumor Hospital.

## Consent to participate statement

Written informed consent was obtained from legally authorized representatives for anonymized patient information to be published in this article.

## Data availability

The authors declare that all data supporting the findings of this study are available within the paper and any raw data can be obtained from the corresponding author upon request.

## Author contribution statement

L Qiao designed the experiments and C Dong carried them out. W Jia analyzed and interpreted the data, and G Sun prepared the manuscript with contributions from all coauthors.
