# Supplementary material for: RAB5A in triple-negative breast cancer: a critical role in macrophage reshaping in an exosomal miR-21-dependent manner
Source: Endocr Relat Cancer. 2024 Apr 12;31(5):e230257. doi: 10.1530/ERC-23-0257 (PMC11046356; doi:10.1530/ERC-23-0257)
Supplement: Supplementary Material [file supplementary_material.pdf]

Supplementary Materials

Supplementary Table 1

Primer sequences used for qRT-PCR

**Gene**	**Sequence (5' to 3')**
RAB5A	F-CAAGAACGATACCATAGCCTAGCAC<br>R-CTTGCCTCTGAAGTTCTTTAACCC
CD163	F-TTTGGACAAGCCGTGACTAGA<br>R-CATTCCCGGTGTTGACATTCC
Arg1	F-GTGGAAACTTGCATGGACAAC<br>R-AATCCTGGCACATCGGGAATC
IL-10	F-CCTCCGTCTGTGTGGTTTGAA<br>R-CACTGCGGTAAGGTCATAGGA
iNOS	F-GTTCCAGATGAATACTGGCAGTC<br>R-GCAACTGAACACTATCTTTCCCT
IL-1β	F-ATGATGGCTTATTACAGTGGCAA<br>R-GTCGGAGATTCGTAGCTGGA
YOD1	F-CTCCCCAAGTGTCATCCCTT<br>R-CTGTGCTTACCAGAACCGTG
FASLG	F-CAAGGTCCATGCCTCTGG AA<br>R-TTGACCAGAGAGAGCTCAGATACG
TGFBI	F-GTGTGTGCTGTGCAGAAGGT<br>R-CATATCCAGGACAGCACTCG
IL12A	F-GATGGCCCTGTGCCTTAGTA<br>R-TCAAGGGAGGATTTTTGTGG
PELI1	F-TGTAGTAACTGACACGGTTCCT<br>R-TCCATCTGATGTCTTCCATTTGG

Supplementary Table 2

Putative miRNAs previously reported involved in macrophage polarization and their corresponding references.

Micro RNAs	Role of miRNAs in macrophage polarization
miR-375	Facilitating the migration and infiltration of tumor-associated macrophage (TAM)[1].
miR-99b	Facilitating the transformation into M1 macrophage[2].
miR-222-3p	Facilitating the M1 macrophages transform to M2 macrophages[3].
miR-511-3p	Suppressing the tumor-promoting effects of TAM[4].
miR-21	Facilitating the TAM transform into tumor-promoting phenotypes[5, 6]; Facilitating the M1 macrophages transform to M2 macrophages[7].
miR-145	Facilitating the M1 macrophages transform to M2 macrophages[8].
miR-125a	Facilitating the M1 macrophages, and suppressing the M2 macrophages[9].
miR-98	Facilitating the TAM transform into tumor-suppressing phenotypes[10]; Facilitating the M2 macrophages transform to M1 macrophages[11].

miR-100	Facilitating the TAM transform into tumor-promoting phenotypes[12].
miR-125b-5p	Facilitating the TAM transform into tumor-promoting phenotypes[13].
miR-203	Facilitating the M2 macrophage[14].
miR-146a-5p	Facilitating the M2 macrophage[15].
miR-28-5p	Facilitating the TAM transform into tumor-suppressing phenotypes[16].
miR-155	Facilitating the M2 macrophages transform to M1 macrophagesM2→M1[17];Facilitating the TAM transform into tumor-suppressing phenotypes[18].
miR-138-5p	Facilitating the M1 macrophages transform to M2 macrophages[19].
miR-340-5p	Facilitating the M2 macrophages transform to M1 macrophages[20].
miR-935	Facilitating the M2 macrophages transform to M1 macrophages[21].
miR-498	Facilitating the M1 macrophages transform to M2 macrophages[22].
miR-940	Facilitating the M1 macrophages transform to M2 macrophages[23].
miR-217	Facilitating the M2 macrophages transform to M2 macrophages[24].
miR-106b-5p	Facilitating the M1 macrophages transform to M2 macrophages[25].
miR-934	Facilitating the M2 macrophages[26].
miR-142-3p	suppressing the M2 macrophages[27].
miR-9	Facilitating the M1 macrophages[28].
miR-195-5p	suppressing the M2 macrophages[29].

References

1. Frank, A.-C., et al., Apoptotic tumor cell-derived microRNA-375 uses CD36 to alter the tumor-associated macrophage phenotype - Nature Communications. Nature Communications, 2019. **10**(1135): p. 1-18

2. Wang, L., et al., Original research: Targeted delivery of miR-99b reprograms tumor-associated macrophage phenotype leading to tumor regression. Journal for Immunotherapy of Cancer, 2020. **8**(2)

3. Ying, X., et al., Epithelial ovarian cancer-secreted exosomal miR-222-3p induces polarization of tumor-associated macrophages. Oncotarget, 2016. **7**(28): p. 43076

4. Squadrito, M.L., et al., miR-511-3p Modulates Genetic Programs of Tumor-Associated Macrophages. Cell Reports, 2012. **1**(2): p. 141-154

5. Sahraei, M., et al., Suppressing miR-21 activity in tumor-associated macrophages

promotes an antitumor immune response. Journal of Clinical Investigation, 2019. **129**(12): p. 5518-5536

6. Xi, J., et al., miR-21 depletion in macrophages promotes tumoricidal polarization and enhances PD-1 immunotherapy - Oncogene. Oncogene, 2018. **37**: p. 3151-3165

7. Hsieh, C.-H., S.-K. Tai, and M.-H. Yang, Snail-overexpressing Cancer Cells Promote M2-Like Polarization of Tumor-Associated Macrophages by Delivering MiR-21-Abundant Exosomes. Neoplasia, 2018. **20**(8): p. 775-788

8. Shinohara, H., et al., Regulated Polarization of Tumor-Associated Macrophages by miR-145 via Colorectal Cancer–Derived Extracellular Vesicles. Journal of Immunology, 2017. **199**(4): p. 1505-1515

9. Zhao, J.-L., et al., Forced activation of notch in macrophages represses tumor growth by upregulating miR-125a and disabling tumor-associated macrophages. Cancer research, 2016. **76**(6): p. 1403-1415

10. Li, L., et al., MiR-98 suppresses the effects of tumor-associated macrophages on promoting migration and invasion of hepatocellular carcinoma cells by regulating IL-10. Biochimie, 2018. **150**: p. 23-30

11. Li, L., et al., MiR-98 modulates macrophage polarization and suppresses the effects of tumor-associated macrophages on promoting invasion and epithelial–mesenchymal transition of hepatocellular carcinoma. Cancer cell international, 2018. **18**(1): p. 1-10

12. Wang, W., et al., miR-100 maintains phenotype of tumor-associated macrophages by targeting mTOR to promote tumor metastasis via Stat5a/IL-1ra pathway in mouse breast cancer - Oncogenesis. Oncogenesis, 2018. **7**(97): p. 1-17

13. Gerloff, D., et al., Melanoma-Derived Exosomal miR-125b-5p Educates Tumor Associated Macrophages (TAMs) by Targeting Lysosomal Acid Lipase A (LIPA). Cancers, 2020. **12**(2): p. 464

14. Takano, Y., et al., Circulating exosomal microRNA-203 is associated with metastasis possibly via inducing tumor-associated macrophages in colorectal cancer. Oncotarget, 2017. **8**(45): p. 78598

15. Yin, C., et al., SALL4-mediated upregulation of exosomal miR-146a-5p drives T-cell exhaustion by M2 tumor-associated macrophages in HCC. OncoImmunology, 2019. **8**(7): p. e1601479

16. Zhou, S.-L., et al., miR-28-5p-IL-34-macrophage feedback loop modulates hepatocellular carcinoma metastasis. Hepatology, 2016. **63**(5): p. 1560-1575

17. Cai, X., et al., Re-polarization of tumor-associated macrophages to pro-inflammatory M1 macrophages by microRNA-155. Journal of Molecular Cell Biology, 2012. **4**(5): p. 341-343

18. He, M., et al., MicroRNA-155 Regulates Inflammatory Cytokine Production in Tumor-associated Macrophages via Targeting C/EBPβ - Cellular & Molecular Immunology. Cellular & Molecular Immunology, 2009. **6**: p. 343-352

19. Xun, J., et al., Cancer-derived exosomal miR-138-5p modulates polarization of tumor-associated macrophages through inhibition of KDM6B. Theranostics, 2021. **11**(14): p. 6847

20. Liu, Y., et al., An miR-340-5p-macrophage feedback loop modulates the progression and tumor microenvironment of glioblastoma multiforme - Oncogene. Oncogene, 2019.

38: p. 7399-7415

21. Zhang, B., et al., INT-HA induces M2-like macrophage differentiation of human monocytes via TLR4-miR-935 pathway. Cancer Immunology, Immunotherapy, 2019. **68**(2): p. 189-200
22. Li, D., et al., miR-498 inhibits autophagy and M2-like polarization of tumor-associated macrophages in esophageal cancer via MDM2/ATF3. Epigenomics, 2021
23. Chen, X., et al., Exosomes derived from hypoxic epithelial ovarian cancer deliver microRNA-940 to induce macrophage M2 polarization. Oncology Reports, 2017. **38**(1): p. 522-528
24. Jiang, B., et al., MiR-217 Inhibits M2-Like Macrophage Polarization by Suppressing Secretion of Interleukin-6 in Ovarian Cancer. Inflammation, 2019. **42**(5): p. 1517-1529
25. Shi, Y., et al., miR-106b-5p Inhibits IRF1/IFN-β Signaling to Promote M2 Macrophage Polarization of Glioblastoma. OncoTargets and therapy, 2020. **13**: p. 7479
26. Zhao, S., et al., Tumor-derived exosomal miR-934 induces macrophage M2 polarization to promote liver metastasis of colorectal cancer. Journal of Hematology & Oncology, 2020. **13**(1): p. 1-19
27. Sonda, N., et al., miR-142-3p Prevents Macrophage Differentiation during Cancer-Induced Myelopoiesis. Immunity, 2013. **38**(6): p. 1236-1249
28. Tong, F., et al., HPV+ HNSCC-derived exosomal miR-9 induces macrophage M1 polarization and increases tumor radiosensitivity. Cancer letters, 2020. **478**: p. 34-44
29. Lin, X., et al., miR-195-5p/NOTCH2-mediated EMT modulates IL-4 secretion in colorectal cancer to affect M2-like TAM polarization. Journal of Hematology &

Oncology, 2019. **12**(1): p. 1-14
